# Have You Been a Victim of COVID-19-Related Cyber Incidents? Survey, Taxonomy, and Mitigation Strategies

**DOI:** 10.1109/ACCESS.2020.3006172

**Published:** 2020-06-30

**Authors:** Saqib Hakak, Wazir Zada Khan, Muhammad Imran, Kim-Kwang Raymond Choo, Muhammad Shoaib

**Affiliations:** 1 Faculty of Computer ScienceUniversity of Northern British Columbia 6727 Prince GeorgeBCV2N 4Z9Canada; 2 Faculty of CS & ISJazan University 123285 Jazan45142Saudi Arabia; 3 College of Applied Computer ScienceKing Saud University 37850 Riyadh11451Saudi Arabia; 4 Department of Information Systems and Cyber SecurityThe University of Texas at San Antonio 12346 San AntonioTX78249USA; 5 College of Computer and Information SciencesKing Saud University 37850 Riyadh11451Saudi Arabia

**Keywords:** COVID-19, cyberattacks, security and privacy, taxonomy, mitigation, potential solutions

## Abstract

Cybercriminals are constantly on the lookout for new attack vectors, and the recent COVID-19 pandemic is no exception. For example, social distancing measures have resulted in travel bans, lockdowns, and stay-at-home orders, consequently increasing the reliance on information and communications technologies, such as Zoom. Cybercriminals have also attempted to exploit the pandemic to facilitate a broad range of malicious activities, such as attempting to take over videoconferencing platforms used in online meetings/educational activities, information theft, and other fraudulent activities. This study briefly reviews some of the malicious cyber activities associated with COVID-19 and the potential mitigation solutions. We also propose an attack taxonomy, which (optimistically) will help guide future risk management and mitigation responses.

## I. Introduction

**FIGURE 1.**
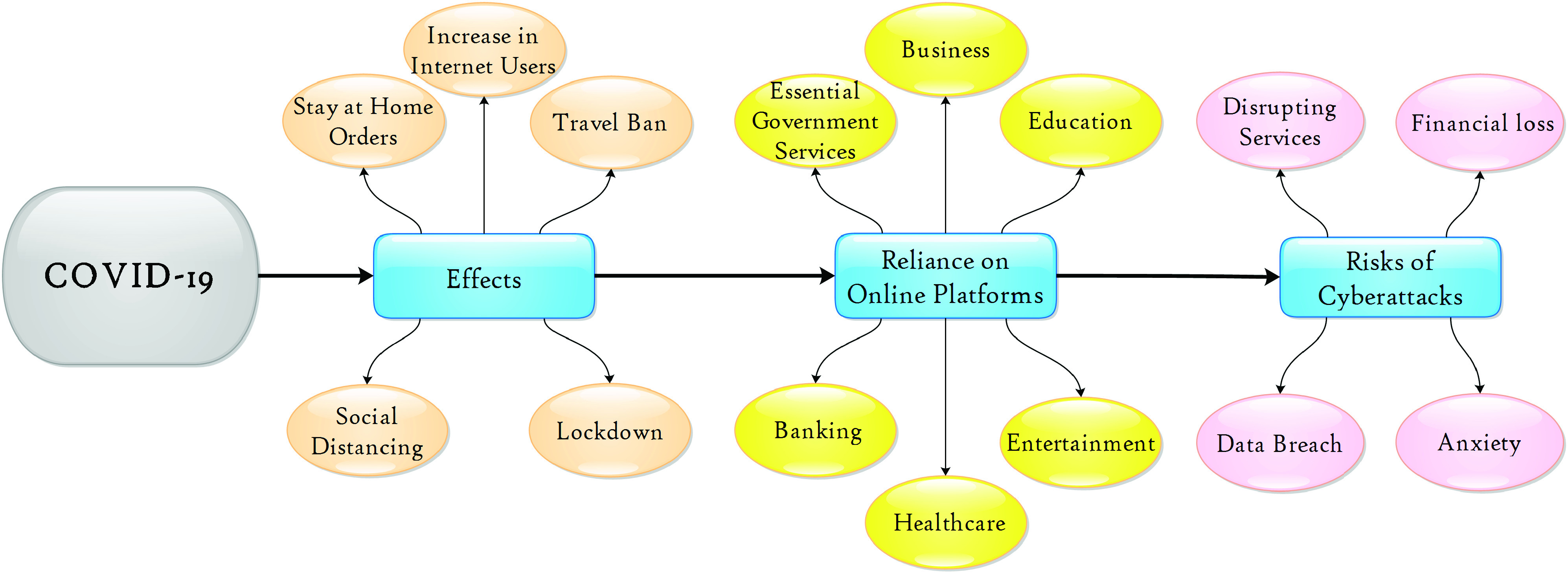
Effects of COVID-19 Pandemic.

COVID-19, which is also referred to as novel coronavirus, 2019-nCoV, or SARS-CoV-2, is among the worst pandemics in recent times and has resulted in numerous countries introducing travel bans, social distancing, lockdowns, and stay-at-home orders [1]. These measures have a broad range of consequences, including those shown in Figure 1. For example, one of the trends is increased remote working and education arrangements, such as using videoconferencing software (e.g., Zoom, Microsoft Teams, and Skype Business) for work and educational purposes.^1^^1^https://www.marketwatch.com/story/zoom-microsoft-cloud-usage-are-rocketing-during-coronavirus-pandemic-new-data-show-2020-03-30, last accessed June 4, 2020.

Corresponding security and privacy risks have also been observed. For example, Singapore’s Minister for Home Affairs indicated that between January and April 2020, “a total of 394 scams related to Covid-19 were reported and victims were cheated of at least SGD 1.4 million”.^2^ The Australian Competition and Consumer Commission’s Scamwatch also reportedly received over 2,700 COVID-19-related scam reports, with an estimated loss of over AUD 16,390,650 as of April 2020.^3^ The US Federal Trade Commission estimated that USD 12 million dollars were lost from COVID-19-related fraudulent activities between January and April 14, 2020, with a total of 18,235 reports related to COVID-19 and up to USD 13.44 million dollars were lost to fraud.^4^ The affected victims range from organizations (e.g., educational and commercial entities), governments, to individuals, such as those listed in Table I. Reports also indicated that urgent surgeries had to be postponed [2]–[4]. However, an extremely challenging endeavor is quantifying the losses (e.g., financial and social) caused by cyberattacks associated with this pandemic, or even fully comprehending the entire threat landscape.^2^https://www.todayonline.com/singapore/close-400-covid-19-related-scams-reported-s14-million-cheated-january-april, last accessed June 4, 2020.^3^https://www.scamwatch.gov.au/types-of-scams/current-covid-19-coronavirus-scams, last accessed June 4, 2020.^4^https://www.consumer.ftc.gov/blog/2020/04/covid-19-scam-reports-numbers, last accessed June 4, 2020.

**TABLE 1:** Examples of COVID-19-Related Cyber Incidents

Sources	Summary
http://www.forbes.com	Hammersmith Medicines Research, a London-based coronavirus vaccine testing facility, was reportedly affected by ransomware. Given that the facility did not pay the ransom amount, personal records of thousands of patients’ information were published online.
http://www.msn.com	Distributed denial of service (DDoS) attacks were launched against the US Health and Human Services departmental servers.
http://www.reuters.com	The World Health Organization (WHO) was reportedly targeted by an advanced persistent threat (APT) actor called DarkHotel, who attempted to steal the passwords of WHO members.
http://www.reuters.com	E-mail accounts of several employees of Monte dei Paschi, an Italian state-owned bank, was reportedly hacked to gain access to sensitive information.
http://www.independent.co.uk	Johns Hopkins University created a map to track global COVID-19 cases, but it was reportedly abused by cyber criminals to infect users and steal their passwords as soon as a user clicks on the map.
http://www.cnet.com	A spyware campaign was reportedly launched through fake applications, such as corona live 1.1, to carry out surveillance activities.

To the best of our knowledge, this study is the first attempt to provide an overview of cyberattacks prevalent during the COVID-19 pandemic. However, possible new attacks could have been perpetrated because the pandemic was still ongoing when this research was being conducted.

This study attempts to map some of these attacks based on categories (see Section III-A). We use these attack categories as bases to discuss potential mitigation strategies (see Section IV). The main contributions of this study are as follows:

• Identify various COVID-19-related cyber threats,
• Develop a new taxonomy of attacks and their effects on security goals, and
• Discuss the potential mitigation strategies to counter the identified threats.

The remainder of this paper is organized as follows. Section 2 briefly reviews the related literature. Section 3 discusses the COVID-19 related cyberattacks prior to the presentation of the taxonomy and potential mitigation strategies in the next section. Sections 4 and 5 present the discussion and conclusion, respectively.

## II. Literature Review

**FIGURE 2.**
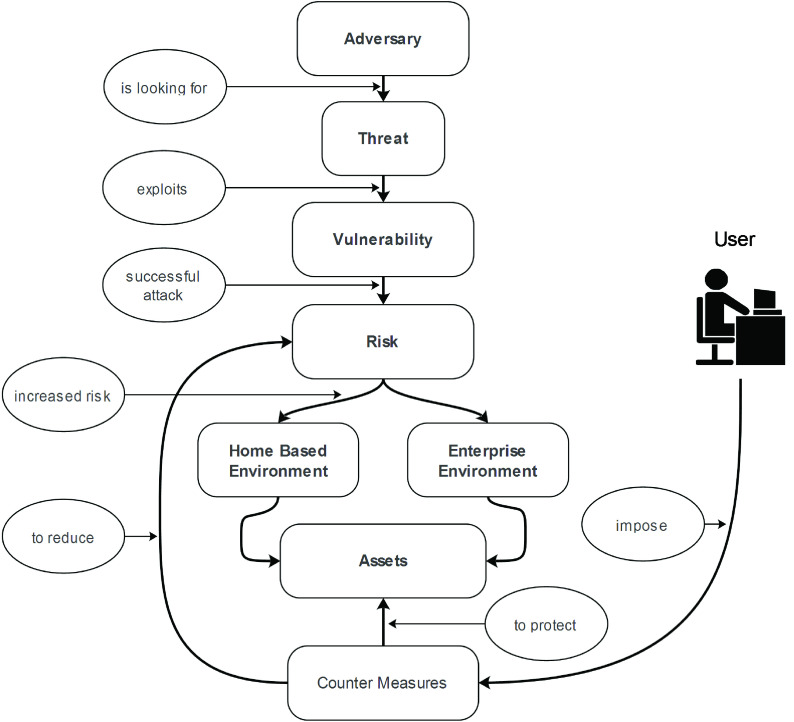
Relationship among threat, vulnerability, and risk.

Cybersecurity is the process of securing assets, networks, programs, and data from any unauthorized access or attack. The evolving nature of attacks makes cybersecurity one of the challenging research areas. To understand information flow within cybersecurity, an important aspect is gaining familiarity with a few key terms, namely, adversary or threat agent, threat, risk, attack, vulnerability, security policy, assets, and countermeasures. Brief descriptions of these terms are provided in Table 2 [5], while the relationship of these terms is presented in Figure 2. Several standard organizations, such as the International Organization for Standardization (ISO) and National Institute of Standards and Technology (NIST), are involved in mitigating the impact of cyberattacks. These organizations are responsible for developing cybersecurity frameworks, security protocols, and guidelines to minimize the impact of attacks. For example, a latest versatile cybersecurity framework proposed by NIST is version 1.1 [6], which is mainly designed for critical infrastructure. A risk management framework was proposed by ISO under standard ISO-31000 [7]. Although several other cybersecurity frameworks are suitable for small and large organizations, the suitability of these frameworks amid the COVID-19 pandemic has yet to be validated. Extensive research should be pursued in this domain, particularly on whether existing cybersecurity frameworks are sufficiently effective to minimize the risks associated with evolving work environments.

**TABLE 2:** Key Security Terms

Terms	Definition
Adversary	Individuals or groups with the aim of carrying out inimical activities
Threat	Any event or situation with the potential of adversely affecting information system sources
Risk	Measure of probability loss resulting from an attack
Attack	Threat carried out by an adversary to collect, disrupt, or damage information system sources
Vulnerability	Any weakness spot within information system resources that can be exploited by an adversary
Security policy	Set of guidelines to maintain the security provisions of an information system resource
Assets	Entity to be protected from attacks and includes hardware, software, data, and networks
Countermeasures	Approaches to mitigate or prevent attacks to secure assets

At present, only few studies have highlighted the effects of COVID-19 in terms of cybersecurity because the majority of the current studies have mostly focused on security, privacy and trust aspects in wireless sensor networks (WSNs) [8], Internet of Things (IoT) [9]–[13], software-defined IoT using edge computing ecosystems [14], smart cities [15], [16], and industrial IoT (IIoT) [17]. However, we were able to find few interesting articles that worked in this direction. Although the majority of the studies have highlighted the implications of tracking applications that violate privacy concerns [18], [19]. One such study has raised concerns in installing the related apps (e.g., TraceTogether, a mobile phone app released by the Singaporean government) [20]. This app works by exchanging tokens with nearby Bluetooth devices. When users are diagnosed with COVID-19, health officials ask users to share such an information via app, thereby possibly leading to different privacy attack, such as simple linkage attack [21].

To date, only a few approaches have been proposed to mitigate privacy concerns. Reference [22] claimed that healthcare data collection is at risk from being compromised by adversaries. To make data collection markedly secure, the authors have proposed a privacy-preservation application called Wetrace, which uses Bluetooth low energy for the message to reach its destination. Reference [23] proposed QUEST, a WiFi-based privacy-preservation technology to track individuals and their interactions. The aforementioned study discussed that existing tracking approaches, such as Bluetooth beacons and smartphone apps, violate individual privacy rights and needs proper privacy-preservation-based approach.

The other studies that highlighted the cybersecurity issues that arised owing to this pandemic include the work of [32], which feature the sectors severely affected by the pandemic and the need for proper security measures to prevent cyberattacks. Similarly, the work of [33] highlighted the cybercrime and cybersecurity challenges that arised from the work-from-home directives from various governments and other organizations. The authors cited the Global Endpoint Security Trend Report and highlighted that approximately 42 percent endpoints worldwide are not secure owing to working from home scenarios, as employees have minimal cybersecurity resources at their disposal. Table 3 presents the other aspects of research to address COVID-19 using information and communication technologies, in which differences between those studies and our research is also highlighted.

**TABLE 3:** Existing COVID-19 Related Studies

	Cyberattacks	Security and Privacy Concerns	Role of Emerging Technologies for Tracking and Monitoring	Prediction and Diagnosis
Z. Allam et al. [2]	✘	✘	✔	✘
H. Cho et al. [20]	✘	✔	✘	✘
A. De Carly et al. [22]	✘	✔	✘	✘
P. Gupta et al. [23]	✘	✔	✘	✘
Z. Yang et al. [24]	✘	✘	✘	✔
B. Pirouz et al. [25]	✘	✘	✘	✔
A. Kumar et al. [26]	✘	✘	✔	✘
L. Wynants et al. [27]	✘	✘	✘	✔
X. Meng et al. [28]	✘	✘	✔	✘
M. Javaid et al. [29]	✘	✘	✔	✘
C. J. Wang et al. [30]	✘	✘	✔	✔
V. Chamola et al. [31]	✘	✘	✔	✘
This Study	✔	✔	✘	✘

## III. COVID-19 Cyber Incidents and Consequences

**FIGURE 3.**
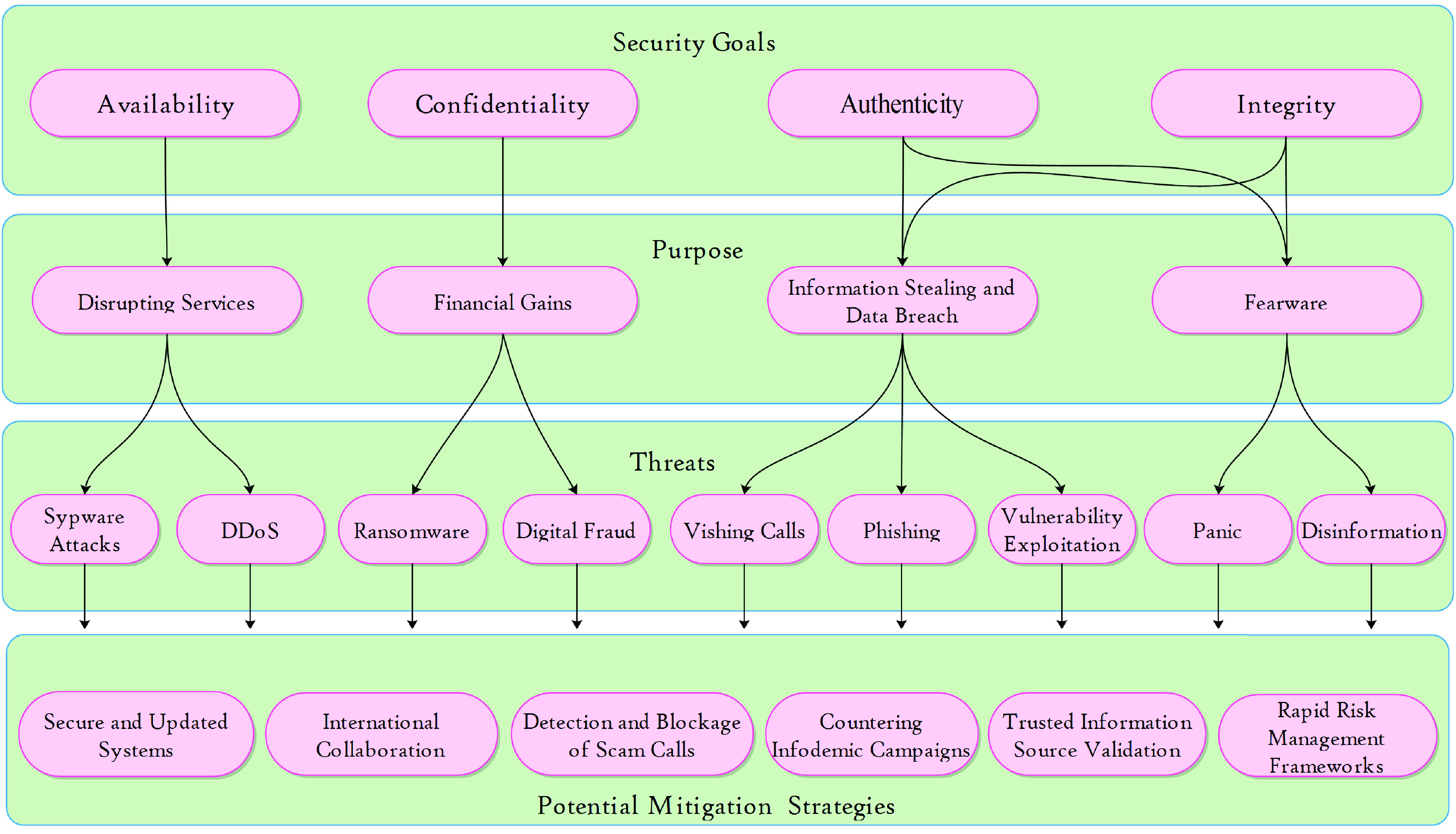
Taxonomy of the COVID-19-themed Cyber incidents.

In this section, we will present the taxonomy of COVID-19 related cyber incidents (see Figure 3), and discuss the associated consequences.

### A. Cyber Incidents

**FIGURE 4.**
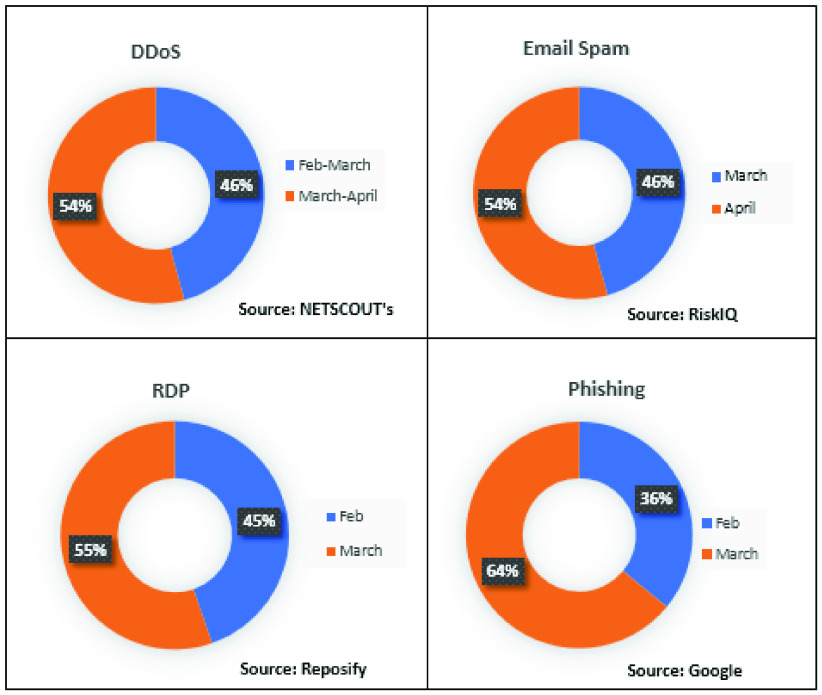
Surge of Cyberattacks amid COVID-19.

Recent statistics have shown that the number of COVID-19-themed cyberattacks has increased in the past weeks and months, as shown in Figure 4. Tables 1 and 4 highlight the popular real-world cybersecurity and malware attacks, respectively, amid the COVID-19 pandemic. These attacks can be broadly categorized on the basis of the intentions of cyber criminals, such as to disrupt essential/entertainment services, obtain illicit financial gain, steal information, and seek to spread fear (see Sections III-A1 to III-A4).

**TABLE 4:** Examples of COVID-19-Themed Malware

Malware	Summary	Sources
Maze	Ransomware	http://www.mcafee.com
Mummy Spider	Utilizes e-mail-thread hijacking techniques to trick victims to download malware samples, such as Emotet.	http://www.crowdstrike.com
AZORult	Information-stealing malware targeting coronavirus online map trackers.	http://www.scmagazine.com
Zloader	Users (tricked into) download(ing) Zloader will result in their system being infected with the Zeus malware.	http://www.zdnet.com
Remote Access Trojan (RAT)	Attempts to take over administrative control of victims’ devices to carry out surveillance or other nefarious activities.	http://www.anomali.com
AndroidOS-ProjectSpy.HRX and IOS-ProjectSpy.A	Steals messages from popular messaging platforms and information related to WiFi and SIM.	http://www.trendmicro.com

#### 1) Disrupting Services

##### a: DDoS Attacks

Europol reported a steady increase in DDoS attacks during the pandemic. These attacks have substantial practical consequences because the number of Internet users also increases owing to social distancing, work-from-home environments, and online educational activities (e.g., video tutorials) [34], among others. An example of such a scenario was reported by the US Health and Human Services Department and occurred in March 2020 [35].

##### b: Spyware Attacks

Spyware is a type of malware used to clandestinely obtain covert information of other systems. This threat has been observed in the current COVID-19 pandemic. For example, COVID-19-related tracker-based apps were reportedly embedded in spyware-based programs to track the activity of users. A popular malicious app is Corona Live 1.1.

#### 2) Financial Gains

##### a: Ransomware Attack

Malware, such as ransomware, are malicious programs designed to facilitate a broad range of nefarious activities [36], [37]. In particular, malware are designed to prevent access to people’s personal data unless a ransom is paid (typically using some cryptocurrency, such as bitcoin). For example, CovidLock, an Android app, was developed to monitor heat map visuals and statistics on COVID-19. Users seeking to install this app have to grant the app certain permissions on the users’ device. As soon as the app is installed, it locks user contacts, pictures, videos, and access to social media accounts. To regain access, users have to pay the ransom using bitcoins. If the ransom is not paid, then users’ information may be published and all data erased from the devices’ memory [38].

##### b: Digital Fraud

Apart from COVID-19-themed malware designed to facilitate illicit financial gains, we also observed an increase in the number of COVID-19-themed gray marketing activities. Examples include attempts to sell personal protective equipment (PPE) or other COVID-19-related products at astronomical prices, or sell counterfeit and unapproved equipment and products. Approximately 2,000 online links were discovered by Interpol and other intelligence agencies between March 3 and 10, 2020 [39]. These links offered to sell COVID-19-related products at considerably high prices. Approximately 13 million Euros worth of pharmaceuticals and 37,000 counterfeit and unauthorized medical devices were reportedly seized during this short period.

#### 3) Information Theft and Data Breach

##### a: Vishing Calls

Telecomputing (e.g., telehealth) is becoming a norm in the current COVID-19 pandemic, in which organizations offer flexible work arrangements to their employees. Given that these employees rely heavily on phone and Internet communications to carry out their business operations, including healthcare advisories, such a communication channel can also be, and have been, exploited by cyber criminals. For example, cyber criminals have been reported to hijack or impersonate business and personal communications via voice phishing (i.e., vishing), robocall scams, and other technical support scams. Cyber criminals have also been reported to abuse voice over IP (VoIP) services to scam individuals into paying for non-existent services or hand over their personal information (e.g., bank account details, social security numbers) [40].

##### b: Vulnerability Exploitation

The existing social distancing requirements have resulted in the closure of such organizations as universities, government agencies, and other non-essential services. This closure has resulted in the significant use of online systems and platforms, such as online learning management systems (LMS) and video conferencing applications and tools (e.g., Zoom). Several incidents, some of which are highly publicized, have been reported, in which cyber criminals identify and exploit vulnerabilities in the aforementioned systems and platforms. One popular but vulnerable platform was reportedly hacked owing to weak security and password mechanism. Consequently, the attackers were able to hijack video conference sessions or gain access to conferencing contents.

##### c: Phishing

Phishing is also a common attack threat observed during the COVID-19 pandemic. RiskIQ [41] reported that over a three-day period (i.e., April 11 to 13, 2020) over 309,000 spam e-mails containing either “corona” or “covid” were discovered. In these e-mails [42], the attackers impersonated the World Health Organization (WHO) or some medical professionals by using such prefixes as “Dr” and “Professor.” These e-mails often contain such subject lines as “COVID-19 updates,” “COVID-19 tracker of your city,” and similar tags designed to lure victims in clicking on the attachment with extensions that include “.rtf” [43].

#### 11) Fearware

##### a: Disinformation

Several infodemic campaigns have also been observed on popular social media platforms, such as Facebook, WhatsApp, and LinkedIn, where fake or misleading information were posted. Examples include claims of ayurvedic medicine being effective against COVID-19 or drinking tea or cow urine can prevent COVID-19 transmission [44]. Although no scientific evidence validate these claims, they created confusion among the public and, in some cases, led to fatalities or injuries. Numerous articles and videos have also been shared through social media platforms that teach how to make home-made hand sanitizers and other related products. There have also been claims on popular social media websites that COVID-19 is not real, and citizens should disregard social distancing requirements. Moreover, COVID-19-themed articles advocating violence against certain ethnicity groups have been reportedly circulating online. Such activities can have fatal consequences.

### B. Effects on Security Goals

All the previously discussed threats serve the same purpose, which is to disrupt security goals and exploit potential vulnerabilities in various sectors, such as health care, entertainment, education, business, banking, and essential government services. The brief descriptions of these security goals and effects are as follows.

#### 1) Confidentiality

Confidentiality ensures that information is accessible only to authorized people and is commonly achieved through encryption, in which information is hidden to the outside world but accessible to participating users. For financial benefits, hackers utilize various type of techniques, such as ransomware, to gain unauthorized access to user devices and encrypt and lock personal files on their mobiles and PCs. These incidents result in considerable financial losses to individuals and organizations [45].

#### 2) Integrity

The main goal of integrity is to safeguard data from any intentional or accidental changes by authorized/unauthorized users [45]. This aspect ensures that information is in its original form and maintains the data consistency of internal and external programs. During the COVID-19 pandemic, a few attacks have focused on the integrity of systems, in which unauthorized health professionals pretend to be authorized professionals and use different approaches (e.g., e-mail spam, phishing calls) to lure users for their malicious financial benefits.

#### 3) Availability

Availability ensures that data and resources are readily available to authorized users, particularly during emergencies [45]. The COVID-19 pandemic has witnessed several attacks that target several sectors (e.g., health care, which was the worst hit) using DDoS and malware attack strategies to disrupt the availability of critical services. The ultimate consequence of compromising this security goal results in rescheduling urgent healthcare surgeries and appointments and delay in chemotherapy, among others.

#### 4) Authenticity

Authenticity [5] is the latest addition to the CIA triad, in which the ultimate goal is to verify that the received message or any data exchange is from that original source only. This objective is often achieved through authentication via static and dynamic authentication methods. Several malware were created during the pandemic to facilitate the stealing of user credentials and information, such as social security numbers [46], [47]. Concerns have also been raised related to privacy and surveillance, such as the use of COVID-19 tracking apps [48].

Figure 3 presents the motivation of attacks, approaches in conducting attacks, and potential mitigation strategies and security goals. The description of mitigation strategies is discussed in the following section.

## IV. Potential Mitigation Solutions

**FIGURE 5.**
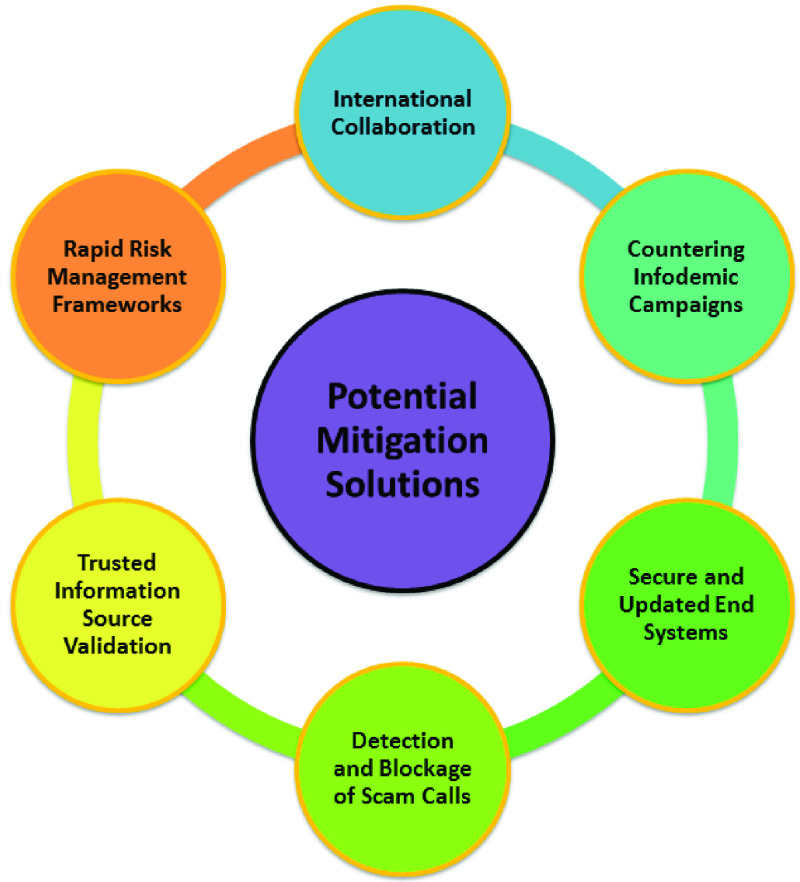
Potential solutions to mitigate cyberattacks during pandemics.

This section provides guidelines for individuals working from home to minimize attacks. We also discuss the potential mitigation approaches to counter future pandemic-themed cyberattacks (see also Figure 5 and Tables 5 and 6).

**TABLE 5:** Potential Solutions and Guidelines

Solutions	Causes	Guidelines
Trusted Information Source Validation	(a) Downloading new low-rated pandemic-related applications, (b) trusting unauthenticated news sources	(a) Evaluate application ratings and reviews, (b) user education for identifying pandemic-related information from trusted and reputable sources, (c) mitigation of ransomware attacks through sophisticated third-party apps
Detection and Blockage of Scam Calls	(a) No single reliable solution available for the detection and mitigation of scam calls, (b) provide personal details through phone and other VoIP-related services	(a) User awareness for identifying and blockage of fraudulent or scam callers, (b) free educational campaigns for not providing any personal and financial information, such as social security number and bank details, through voice calls, (c) avoid or disregard free offers for pandemic testing and vaccination
International Collaboration	(a) Lack of international collaboration to combat pandemic-themed cyberattacks	(a) Establishment of an international task force to facilitate the sharing of current cyber threat intelligence (e.g., threat vectors and techniques), (b) international cyber hygiene educational and training programs, (c) financial support from such organizations as the International Monetary Fund (IMF) can be used to develop tools and skills to mitigate these cyber threats
Countering Infodemic Campaigns	(a) Fake information spreading through social media for panic and financial gains	Identification and classification of fake or misleading news through human-in-loop machine learning techniques.
Secure and Updated Systems	Increase of system usage at home owing to social distancing	(a) Patching of operating systems and applications, (b) free of charge reliable security products (e.g., anti-malware and anti-viruses) during pandemics

**TABLE 6:** Security Guidelines for Users Working From Home

Tips	Reasons
Increase your awareness related to cyberattacks	Extensive information is available to equip individuals, whether new or technical computer users, with the necessary and basic cybersecurity knowledge. Such information as creating strong passwords, identifying vulnerable malware links, and using social media wisely, can help users mitigate numerous cyberattacks. A few of the related popular guidelines are available on ^1^, ^2^, ^3^.
Update installed anti-virus and anti-malware products through original vendors	Given that attacks evolve over time, anti-malware products should be updated to quarantine/counter the effects of new attacks. Different strategies to update anti-virus products are provided by ^4^.
Be cautious to e-mails from unfamiliar sources and the following categories: promotional/special offers, surveys or announcements of any kind, charity-based, bank-related and employers.	These malicious e-mails crafted by scammers encourage users to provide personal information by clicking on links and downloading attachments, and lure users through lucrative offers, such as free entertainment subscriptions, lottery tickets, and cash rewards. The intention is either to damage the system or steal money.
Consistently back-up data	In worst-case scenario of data being compromised, corrupted, or stolen, backing up your data to external devices, such as USBs and hard disks, is recommended.
Do not provide bank/personal details via phone/email for any of the system maintenance services	In the majority of cases, new computer users are tricked by scammers through telephone calls or e-mails. They pretend to update the host system remotely with the intention of hacking it and stealing bank account details.
Be vigilant while clicking online meeting platform links, such as Zoom, Google Meets, and Microsoft Teams	Attackers can impersonate such links as well. A recent example in which a victim pretending it to be from Microsoft teams clicked the following link ^5^ and ended up downloading malware. There are also fake Google Meets domains, such as ”Googelmeetscom.” Further guidelines to mitigate this attack is provided by ^6^.
Use virtual private network (VPN)	VPN provides a private tunnel for users, in which information is encrypted and cannot be accessed by hackers. Hence, organizations can secure the home networks of employees using VPN.
Consistently shutdown laptop or home computer	Some software updates, such as firewall settings and Windows-patch updates, require system restart to be effective. Moreover, system shutdown flashes temporary and unimportant data and stops memory leaks.
Change passwords frequently	A good practice for employees is to frequently change their passwords while accessing online services from their homes. This practice can substantially reduce the impact of passive attacks.
Avoid public WiFi spots	Never use public WiFi spots to access information of your organization or any banking related transactions owing to unencrypted network traffic and legitimacy of these spots.
Strictly follow bring-your-own-device (BYOD) policy	Organizations that allow employers to use their own devices for work provide BYOD policies. These policies include certain security guidelines that aid employees secure their respective devices. Further general guidelines on protecting information while working from home can be found at ^7^.

^1^ https://www.comtact.co.uk/blog/6-steps-of-a-successful-cyber-security-user-awareness-programme

^2^ https://www.iiroc.ca/industry/Documents/CybersecurityBestPracticesGuide_en.pdf

^3^ https://www.enisa.europa.eu/publications/archive/copy_new-users-guide/at_download/fullReport

^4^ https://www.us-cert.gov/sites/default/files/recommended_practices/Recommended Practice Updating Antivirus in an Industrial Control System_S508C.pdf

^5^ http://loginmicrosoftonline.com-common-oauth2-eezylnrbmedyacamcom/common/oauth2/

^6^ https://www.us-cert.gov/ncas/current-activity/2020/04/02/fbi-releases-guidance-defending-against-vtc-hijacking-and-zoom

^7^ https://www.oipc.bc.ca/guidance-documents/1447

### A. Trusted Information Source Validation

One of the potential approaches to mitigate ransomware attacks is to vet third-party apps and educate users, thereby enabling them to identify trusted or reputable sources (e.g., government organizations or reputable research and healthcare institutions). App ratings can also be another indication whether apps are trustworthy. However, this approach will not work for new apps, particularly in pandemic-type situations.

### B. Detection and Blockage of Scam Calls

VoIP service providers can play an effective role in mitigating scam call threats, such as assisting to raise user awareness and actively identify and block potentially fraudulent or scam callers (e.g., based on red flag indicators, such as robot calls). Although not all users are cyber aware, free educational campaigns, such as not sharing personal information through voice calls and disregarding online offers that are too good to be true (e.g., free medical tests and vaccinations), could be intensified during pandemics.

The other possible mitigation strategy involves the design and development of anti-spam detectors based on artificial intelligence (AI). Using the data from previous pandemics, an AI-based bot can be developed to answer calls (instead of users) and verify whether an incoming call is a spam or not.

### C. International Collaboration

Evidently, we need collective effort from different countries and governments during pandemics, such as the current COVID-19 emergency. To combat pandemic-themed cyber threats, effort and countermeasures are required from the international community, including the establishment of an international task force to facilitate the sharing of current cyber threat intelligence (e.g., threat vectors and techniques).

The importance of financial support cannot be understated in international collaboration activities (e.g., cyber hygiene education). However, many other competing priorities are present during pandemics. Hence, the support of the community and international organizations should be sought to fund mitigation initiatives. For example, financial support from such organizations as the International Monetary Fund (IMF) can be used to develop tools and skills to mitigate cyber threats.

### D. Countering Infodemic Campaigns

To counter infodemic campaigns, we need the support and involvement of a broad range of stakeholders, such as social media platforms. However, determining whether posted contents are fake can be challenging, particularly when relating to ongoing pandemics. Hence, computer and social scientists and healthcare professionals have roles in collaborating and designing approaches (e.g., based on human-in-the-loop machine learning techniques) to considerably identify and classify fake or misleading news.

### E. Secure and Updated Systems

Given the increased use of systems at homes due to social distancing measures, effort should be exerted to ensure that home systems are patched and secure. For example, patching operating systems and applications is one of the key cyber mitigation strategies recommended by the Australian Signals Directorate’s Australian Cyber Security Centre [49].

Security organizations can also play a role, such as by not charging subscriptions for their security products (e.g., anti-malware software) during pandemics.

### F. Rapid Risk Management Frameworks

Risk management framework is an effective method to access, mitigate, and evaluate risks associated with the threat. Several risk management frameworks are available such as for scada systems [50], online services [51], and cyber physical systems [52]–[54]. Accordingly, a pandemic such as COVID-19 warrants new and rapid framework that can be implemented immediately. Such a framework should be robust, scalable, time-efficient, and accurate which can be easily followed by technical/non-technical computer experts within dynamic environments whether home- or office-based environment.

## V. Discussion

The most pronounced impact of COVID-19 is the shift of the cyber security landscape from an enterprise to a home environment. The fortuitous shift has provided many new opportunities to hackers and cybercriminals, thereby resulting in an increased risk of vulnerability exploitation. During the COVID-19 pandemic, a new wave of cyberattacks was recorded. Working from home has increased the risk of cyberattacks owing to various reasons, which is highlighted in Figure 3. In the enterprise or corporate environment, the security of all assets (hardware and software) are properly managed by the IT support staff and access to systems, and the internet is governed under strict cybersecurity policies and SOPs. IT-related assets are patched and updated regularly. However, working from home using employees own devices with their unsafe networks increase the opportunities of cyber threats. Accordingly, working with these unprotected and unsecured communication channels from home provides an entry point to hackers and cybercriminals.

User awareness is critical to mitigate and reduce the risk of such cyberattacks in the future. We summarized the key user awareness guidelines in Tables 5 and 6 that are suitable for home-based environment and vice versa. The most important security guidelines are as follows. First, organizations that allow employees to use their own devices to work from home provide BYOD policies, which contain security guidelines that aid employees to secure their respective devices. Second, VPNs should be used while working from home to communicate between employee personal devices and enterprise systems. Lastly, the cybersecurity awareness of employees should be enhanced regularly through cybersecurity education and training programs. Gamification [55] may be explored to further motivate people to gain cybersecurity awareness. The need to include basic cybersecurity curriculum in medical education and for a dynamic cybersecurity risk management framework should be highlighted to cope with pandemics.

Emerging technologies (e.g., AI, machine learning, IoT, IIoT, Industry 4.0, blockchain, Fog, edge computing [56], and mobile and wireless technologies) have extremely important roles in addressing pandemics, such as COVID-19, specifically relate to tracking/monitoring COVID-19 patients, infected areas, pandemic spreading prediction, expediting the development process of new vaccines for COVID-19, and diagnosing COVID-19.

## VI. Concluding Remarks

This study explored COVID-19 themed cyberattacks and categorized them into four categories: disrupting services, financial gains, information theft, and fearware, and further categorized into sub-categories (e.g., malware, ransomware, phishing). We used these categories to present potential mitigation solutions. The cyberattack taxonomy and potential mitigation strategies can also facilitate cyberattack prevention effort plannings in future pandemics. In the future, we intend to extend the proposed taxonomy and propose risk management model for these pandemics.
